# Recommender-based bone tumour classification with radiographs—a link to the past

**DOI:** 10.1007/s00330-024-10672-0

**Published:** 2024-03-15

**Authors:** Florian Hinterwimmer, Ricardo Smits Serena, Nikolas Wilhelm, Sebastian Breden, Sarah Consalvo, Fritz Seidl, Dominik Juestel, Rainer H. H. Burgkart, Klaus Woertler, Ruediger von Eisenhart-Rothe, Jan Neumann, Daniel Rueckert

**Affiliations:** 1 Department of Orthopaedics and Sports Orthopaedics, Klinikum rechts der Isar, Technical University of Munich, Munich, Germany; 2 Institute for AI and Informatics in Medicine, Technical University of Munich, Munich, Germany; 3 Institute of Biological and Medical Imaging, Helmholtz Zentrum München, Neuherberg, Germany; 4 Institute at Helmholtz: Institute of Computational Biology, Oberschleißheim, Germany; 5 Chair of Biological Imaging at the Central Institute for Translational Cancer Research (TranslaTUM), School of Medicine, Technical University of Munich, Munich, Germany; 6 Musculoskeletal Radiology Section, Klinikum rechts der Isar, Technical University of Munich, Munich, Germany

**Keywords:** Bone neoplasms, Deep learning, Classification, Machine learning, Radiography

## Abstract

**Objectives:**

To develop an algorithm to link undiagnosed patients to previous patient histories based on radiographs, and simultaneous classification of multiple bone tumours to enable early and specific diagnosis.

**Materials and methods:**

For this retrospective study, data from 2000 to 2021 were curated from our database by two orthopaedic surgeons, a radiologist and a data scientist. Patients with complete clinical and pre-therapy radiographic data were eligible. To ensure feasibility, the ten most frequent primary tumour entities, confirmed histologically or by tumour board decision, were included. We implemented a ResNet and transformer model to establish baseline results. Our method extracts image features using deep learning and then clusters the *k* most similar images to the target image using a hash-based nearest-neighbour recommender approach that performs simultaneous classification by majority voting. The results were evaluated with precision-at-*k*, accuracy, precision and recall. Discrete parameters were described by incidence and percentage ratios. For continuous parameters, based on a normality test, respective statistical measures were calculated.

**Results:**

Included were data from 809 patients (1792 radiographs; mean age 33.73 ± 18.65, range 3–89 years; 443 men), with Osteochondroma (28.31%) and Ewing sarcoma (1.11%) as the most and least common entities, respectively. The dataset was split into training (80%) and test subsets (20%). For *k* = 3, our model achieved the highest mean accuracy, precision and recall (92.86%, 92.86% and 34.08%), significantly outperforming state-of-the-art models (54.10%, 55.57%, 19.85% and 62.80%, 61.33%, 23.05%).

**Conclusion:**

Our novel approach surpasses current models in tumour classification and links to past patient data, leveraging expert insights.

**Clinical relevance statement:**

The proposed algorithm could serve as a vital support tool for clinicians and general practitioners with limited experience in bone tumour classification by identifying similar cases and classifying bone tumour entities.

**Key Points:**

• *Addressed accurate bone tumour classification using radiographic features.*

• *Model achieved 92.86%, 92.86% and 34.08% mean accuracy, precision and recall, respectively, significantly surpassing state-of-the-art models.*

• *Enhanced diagnosis by integrating prior expert patient assessments.*

**Graphical abstract:**

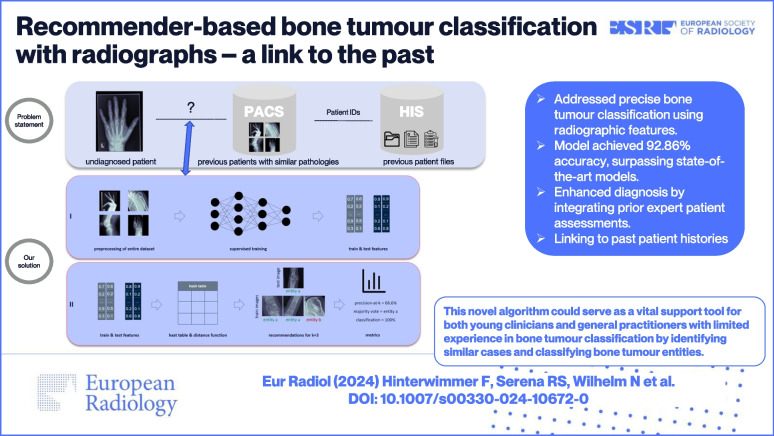

**Supplementary Information:**

The online version contains supplementary material available at 10.1007/s00330-024-10672-0.

## Introduction

Bone tumours are a group of rare and diverse types of neoplasms [1–4]. The vast majority of primary bone tumours are benign, whereas malignant primary bone tumours account for 0.2% of all malignancies in adults [3, 5]. It is crucial to diagnose bone tumours early, as this directly affects the patient’s prognosis and curability [1]. Hence, prompt referral to a specialised tumour centre to determine tumour malignancy, establish a specific diagnosis and initiate early treatment is essential [6]. Unfortunately, delays of more than 1 year often occur in clinical practice, partly due to the lack of specific symptoms in the early stages and the fact that non-oncologically trained orthopaedic surgeons [4, 7, 8], primary care physicians or paediatricians only encounter about three malignant musculoskeletal (MSK) tumours in their professional career and therefore lack the experience in unequivocally identifying these complex tumour entities [7].

Imaging is crucial in diagnosing bone tumours [5]. The Musculoskeletal Tumor Society and American Academy of Orthopedic Surgeons recommend radiographs as the initial screening tool [5, 8]. While CT and MRI provide additional diagnostic information, they should not delay initial medical care [5]. Definitive diagnosis typically requires a combination of imaging, histopathologic findings and clinical presentation, with further detailed imaging assessments recommended at specialised MSK tumour centres [9].

Diagnostic imaging is rapidly advancing with significant technological and market growth, leading to an increase in imaging data [10–12]. In MSK radiology and orthopaedic oncology, precision medicine and image interpretation are increasingly critical. Despite the growing use of artificial intelligence (AI) and deep learning (DL) in cancer research, their application in MSK tumour research remains limited [2, 13]. However, these advanced data analysis techniques hold promise for revolutionising MSK tumour diagnostics and enhancing healthcare delivery [14].

As AI technologies evolve, various medical imaging applications are being developed, often focusing on comparing AI’s performance with that of human experts in tasks like pathology classification [15–17]. Among these, recommender systems (RS) offer a novel approach, primarily suggesting options based on user preferences, bypassing extensive algorithm training [18]. While traditionally used in commercial settings, RS are increasingly recognised for their potential in medical decision-making, such as recommending drug therapies or identifying similar patient cases based on medical history and imaging data [19, 20].

MSK tumour centres have extensive knowledge and experience lying dormant in their hospital information system (HIS) and picture archiving and communication system (PACS) based on patients treated for MSK tumours in the past. In this study, we present a DL-based algorithm that recommends similar patients based on clustering of radiographic features, draws on the extensive experience dormant in clinical systems based on previous patient histories and simultaneously classifies multiple bone tumour pathologies to enable early and specific diagnosis.

## Materials and methods

The local institutional review and ethics board approved this retrospective study (no. 48/20S). The study was performed in accordance with national and international guidelines. Informed consent was waived for this retrospective and anonymised study. The general structure of the manuscript follows the *Checklist for artificial intelligence in medical imaging* (CLAIM [21]).

### Eligibility criteria

**Fig. 1:**
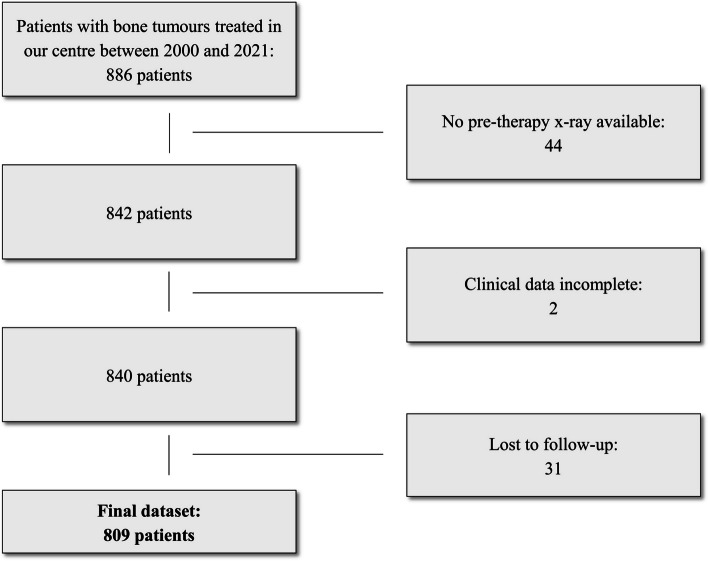
Flow diagram showing the application of eligibility criteria to create a final dataset

For this single-centre study, we conducted a search through the database of our MSK tumour centre. All patients treated for primary bone neoplasms (based on the according ICD codes) between 2000 and 2021 were screened. Patients with the following primary tumours were selected, as these are the most frequent ones in our database: aneurysmal bone cyst (ABC), chondroblastoma, chondrosarcoma, enchondroma, Ewing sarcoma, fibrous dysplasia, giant cell tumour, non-ossifying fibroma (NOF), osteochondroma and osteosarcoma. The diagnosis of malignant lesions was verified by histopathology as standard of reference. Benign and intermediate lesions were either verified by histopathology, if available, or discussed in the local tumour board and classified according to radiological features known from the literature [22]. The clinical and imaging data were retrieved from our HIS and PACS, respectively. To ensure the feasibility of the proposed model, the ten most frequent entities were considered. Any tumour representation in the radiographs was eligible. Forty-four patients with inadequate imaging (no pre-operative/pre-therapy radiographs), two patients with incomplete clinical data and 31 patients lost to follow-up were excluded. Subsequently, 809 patients with 1792 respective radiographs were found (Fig. 1). The curation and validation of the data were conducted by two orthopaedic residents (S.C., S.B.) and a senior MSK radiologist (J.N.), respectively, with support of a data scientist (F.H.).

### Demographics and statistical evaluation

Descriptive data is presented according to the *Strengthening the Reporting of Observational studies in Epidemiology* (STROBE [23]) guidelines. Discrete parameters were described by incidence and percentage ratios. For continuous parameters, based on a Shapiro-Wilk normality test, respective statistical measures were computed.

The mean classification accuracy, precision and recall of the baseline models are calculated based on their performance on the test data. In our multiclass setting, classification accuracy is calculated as the ratio of correctly predicted instances to the total number of test data instances. Precision and recall are measured for each class individually and then averaged: precision is the ratio of true positive predictions of each class to all predictions made for that class, and recall is the ratio of true positive predictions of each class to all actual instances of that class in the test data. The RS clustering results are assessed using a precision-at-*k* metric, which calculates the proportion of relevant items within the top-k recommendations. To compute the final classification accuracy, precision and recall of the proposed model, we compared the correct predictions obtained through a majority vote from the *k*-closest images in the RS against the labels of the respective target images in the test data. About 10% of the total dataset represents external imaging data obtained from other institutions and integrated into our Health Information System (HIS) and Picture Archiving and Communication System (PACS). The dataset is divided into training (80%) and test data (20%), with the metrics being calculated solely on the test data. This test subset exclusively contains patients with a single image to avoid any overlap with the training dataset. The dataset was stratified based on the types of bone tumours, ensuring that each tumour type was proportionally represented in both the training and test subsets. The final metrics, including classification accuracy, precision and recall, were determined three times using randomly shuffled data, and the corresponding mean values were calculated. In addition, the normality of the distribution of performance results was assessed. Based on the outcome of normality tests, suitable statistical methods were chosen to evaluate the significance of model performance metrics.

### Model training

Model training and inference was conducted on a DGX Station A100 with four 80 GB graphical processing units (Nvidia Corporation), 64 2.25 GHz cores and 512 GB DDR4 system memory running on a Linux/Ubuntu 20.04 distribution (Canonical). Preprocessing and model implementation were performed in Python 3.11.1 (https://www.python.org/) using PyTorch 1.13.1 and cuda toolkit 12.0 (https://pytorch.org/).

### Algorithm

**Fig. 2:**
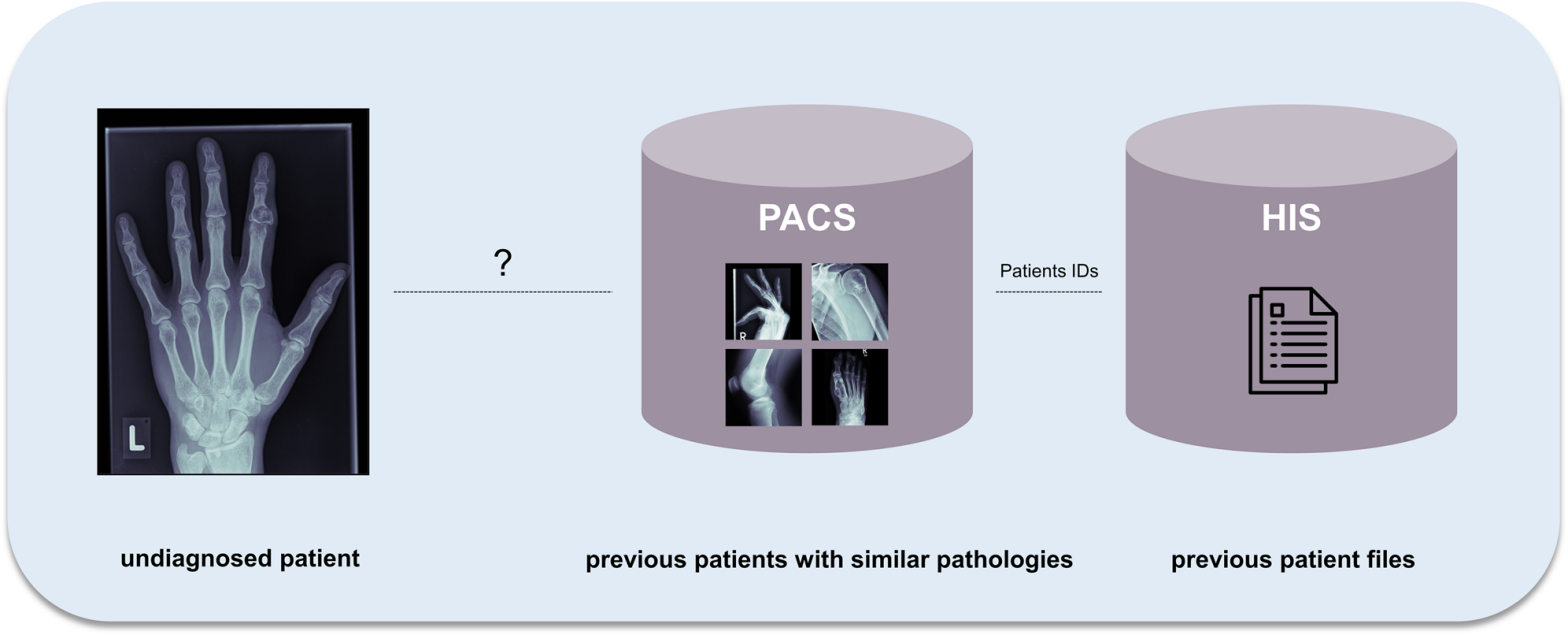
General concept of the proposed method—clustering new patients with previous patients based on radiographs to identify similar cases and classify tumour entity (PACS, picture archiving and communications systems; HIS, hospital information system)

**Fig. 3:**
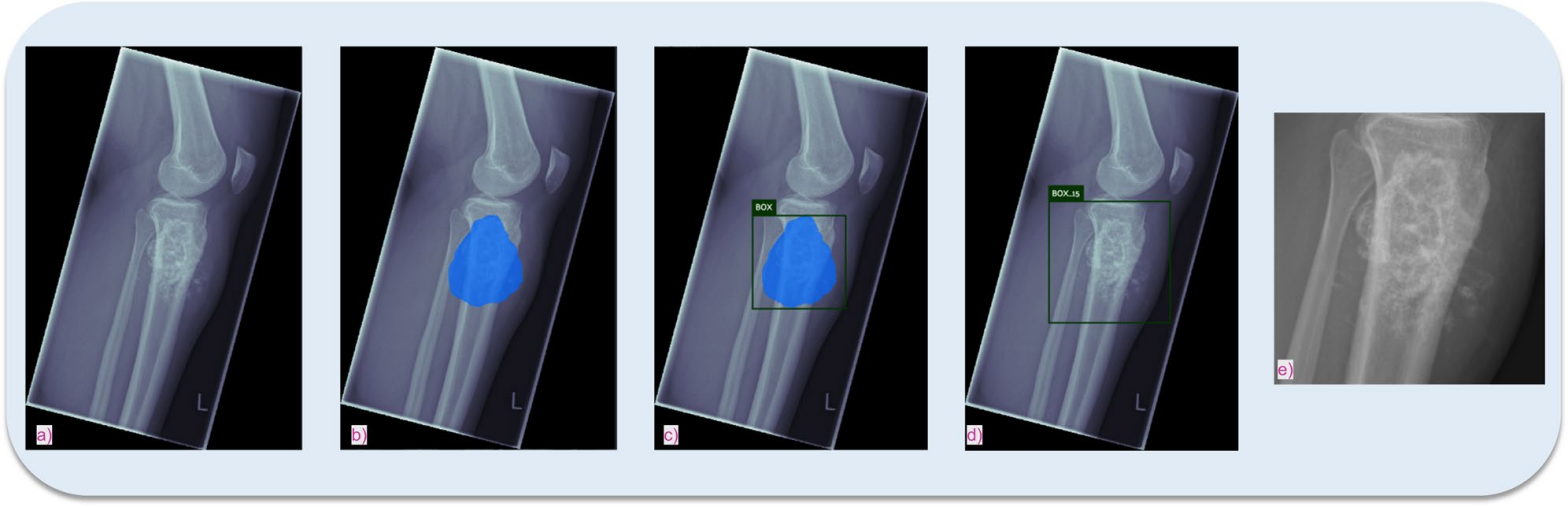
Exemplary creation of bounding boxes focusing the tumourous tissue by the segmentation algorithm of Bloier et al [26]: (**a**) initial image, (**b**) segmented tumour, (**c**) calculated bounding box, (**d**) bounding box with 15% margin to assure all tumour tissue is captured, (**e**) cropped image

**Fig. 4:**
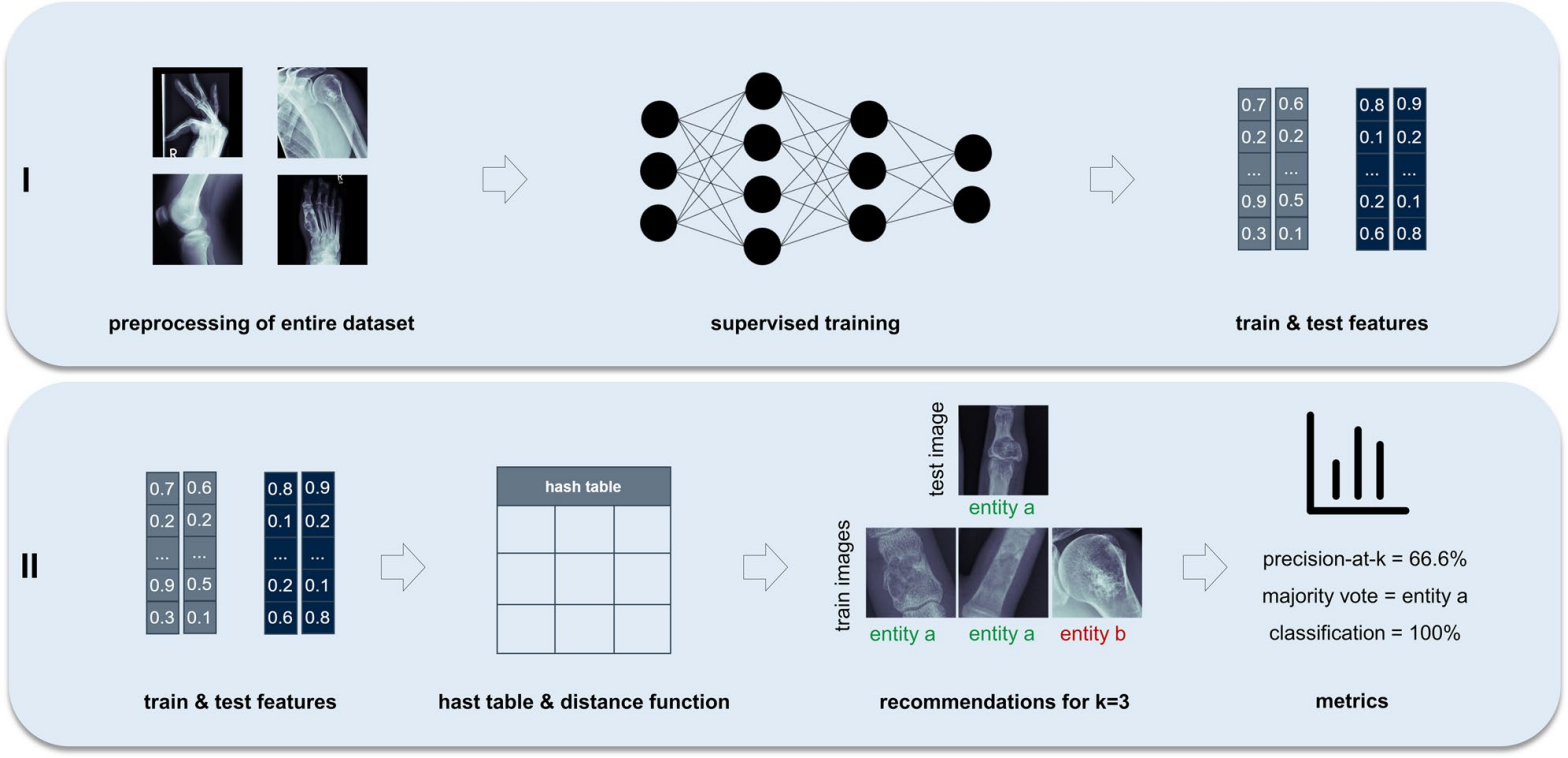
Flow chart of the proposed model—(**I**) preparing the images, training of the convolutional neural network, saving the model and features; (**II**) calculating the high dimensional distances with a distance function, adding a hash tables, clustering of the most similar x-rays and calculating a precision-at-*k* and a tumour entity classification with a majority vote of the *k*-clustered images

The general concept of the proposed framework is shown in Fig. 2: identification of the most similar cases from previous patients based on radiographs with respect to an undiagnosed image. First, to create baselines for bone tumour entity classification, we calculated classification metrics by straightforward application of a standard [24] (baseline 1) and a state-of-the-art [25] (baseline 2) DL model to a multi-entity classification task. For the implementation of our proposed approach, we performed two main steps: (**I**) to emphasise on tumourous tissue rather than background or non-relevant tissue, we created bounding boxes around the region of interest, which can be accomplished algorithmically [26] or through manual cropping by a domain expert (Fig. 3). We employ the model from baseline 1. The trained model as well as the extracted features from the training data was saved. After training was completed, we calculated the image features of the test data by running the data through the trained convolutional neural network model. (**II**) We created a hash table. Instead of comparing each set of new image features to the training data features, we used locality-sensitive hashing (LSH), an approximate nearest neighbour algorithm that reduces the computational complexity from O(N^2^) to O(log N). LSH generates a hash value for image features by taking the spatiality of the data into account. Data elements that are similar in high dimensional space have a higher chance of obtaining the same hash value [27]. Based on a hamming distance function, we computed the *k*-nearest neighbours with respect to each target image. By assigning the *k*-nearest neighbours (from training images) to one cluster along with the target image (test image), we established a link between the undiagnosed patient and past patient cases stored in our database. Since local patient identifiers from the training data patients are known, this allowed us to potentially link to experiences from previous patients in our clinical systems, e.g. radiology reports, laboratory results and therapy results. Furthermore, we obtained a classification of tumour entities by applying a majority vote to the entities of the images clustered to the target image. Figure 4 illustrates the proposed approach.

## Results

### Dataset

The mean age of patients was 33.73 with a standard deviation of 18.65 and a range of 3 to 89. Osteochondroma was the most common entity, accounting for 28.31% of the total dataset, while Ewing sarcoma was the least frequent entity representing only 1.11% of the dataset. The gender was close to similarly distributed (males 54.76%, females 45.24%) with a slight tendency towards males. The most frequent location of tumour occurrence was the femur with 36.71%, while a tumour only occurred once at the os sacrum representing 0.12% of the whole dataset. Further details of the continuous and discrete characteristics are displayed in Table 1.

**Table 1:** Distribution of continuous and discrete characteristics (*IQR* interquartile range)

Characteristic	Shapiro Wilk test	Median	IQR	#	%
Patients					
Age	*W*(809) = 0.94, *p* < .001	30.00	30.00	–	–
Entity					
Aneurysmal bone cyst (ABC)	–	–	–	49	6.06%
Chondroblastoma	–	–	–	18	2.22%
Chondrosarcoma	–	–	–	124	15.33%
Enchondroma	–	–	–	181	22.37%
Ewing sarcoma	–	–	–	9	1.11%
Fibrous dysplasia	–	–	–	31	3.83%
Giant cell tumour	–	–	–	51	6.30%
Non ossifying fibroma (NOF)	–	–	–	33	4.08%
Osteochondroma	–	–	–	229	28.31%
Osteosarcoma	–	–	–	84	10.38%
Gender					
Female	–	–	–	366	45.24%
Male	–	–	–	443	54.76%
Location	–	–	–		
Clavicula	–	–	–	7	0.87%
Columna vertebralis	–	–	–	4	0.49%
Femur	–	–	–	297	36.71%
Fibula	–	–	–	42	5.19%
Humerus	–	–	–	124	15.33%
Manus	–	–	–	62	7.66%
Os ilium	–	–	–	24	2.97%
Os ischii	–	–	–	8	0.99%
Os pubis	–	–	–	11	1.36%
Os sacrum	–	–	–	1	0.12%
Patella	–	–	–	7	0.87%
Pes	–	–	–	42	5.19%
Radius	–	–	–	12	1.48%
Scapula	–	–	–	17	2.10%
Tibia	–	–	–	146	18.05%
Ulna	–	–	–	5	0.62%

### Model performances

For both baseline models, we conducted extensive hyperparameter tuning to optimise their performance as well as a fivefold cross-validation. Key hyperparameters adjusted included learning rate, batch size and number of training epochs. Additionally, we employed several data augmentation techniques (rotations, horizontal and vertical flipping) to enhance the dataset and prevent overfitting. The optimised values for each hyperparameter were as follows: learning rate 0.003/0.0025, batch size 8/8 number of training epochs 85/77 and probability for applying data augmentation 0.3/0.3 respectively for the ResNet and the transformer model. We accomplished a mean test accuracy/precision/recall of 54.10%, 55.57% and 19.85% with a pretrained ResNet32 [24] model and 62.80%, 61.33% and 23.05% with a state-of-the-art Vision Transformer model [25, 28] for classifying the tumour entities on a test split. For our proposed method, the respective precision-at-*k* for *k* = 1/3/5/7 was 65.46%/62.58%/62.06%/61.48%. The classification metrics based on the described majority vote on the clustered images was 65.46%/92.86%/92.13%/92.01%. For *k*, only odd values were used to facilitate meaningful calculation of the majority vote. No higher value than seven was chosen because the lowest number of entity samples was only nine (Ewing sarcoma) and, therefore, considering only odd values, a maximum of seven samples could be assigned. Table 2 displays the results for the two baseline models as well as the result of the best configuration.

**Table 2:** Tumour entity classification results—mean of accuracy, precision and recall with standard deviation

	Baseline 1: ResNet50	Baseline 2: Transformer	**Our approach**
Accuracy			
Training	77.01 ± 1.11	82.37 ± 2.20	–
Validation	58.69 ± 3.04	69.54 ± 2.87	–
Test	54.10 ± 2.91	62.80 ± 1.90	**92.86 ± 0.59**
Precision			
Training	79.10 ± 0.76	80.44 ± 2.29	–
Validation	60.23 ± 2.09	67.91 ± 1.39	–
Test	55.57 ± 2.00	61.33 ± 2.10	**92.86 ± 0.59**
Recall			
Training	28.26 ± 0.41	30.23 ± 0.99	–
Validation	21.53 ± 1.12	25.52 ± 0.81	–
Test	19.85 ± 1.07	23.05 ± 0.70	**34.08 ± 2.76**

**Fig. 5:**
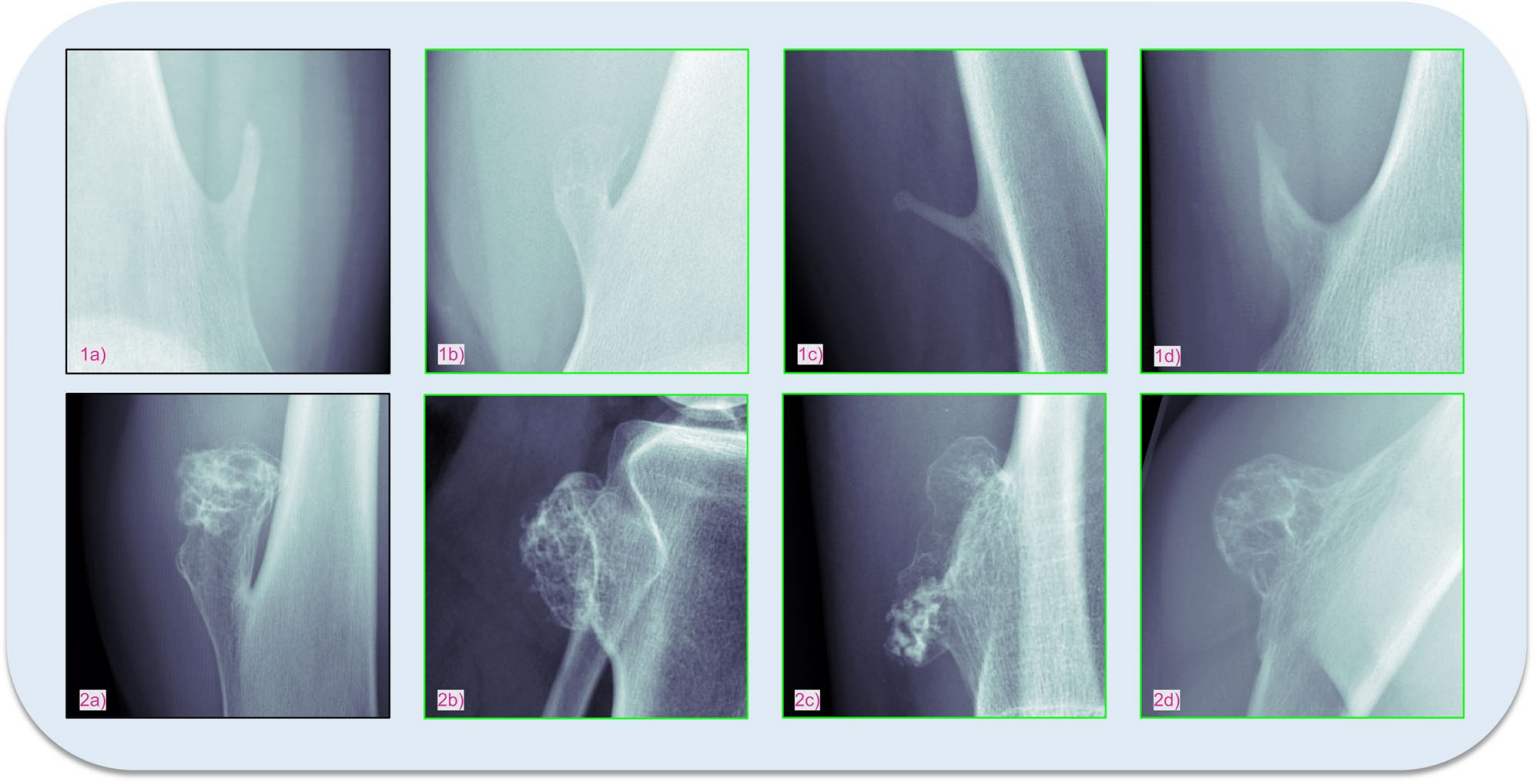
Examples of osteochondroma X-rays showcasing the model’s ability to accurately cluster different appearances of the same tumour entity. The target image is marked with a black frame, while correctly matched images are highlighted with a green frame

**Fig. 6:**
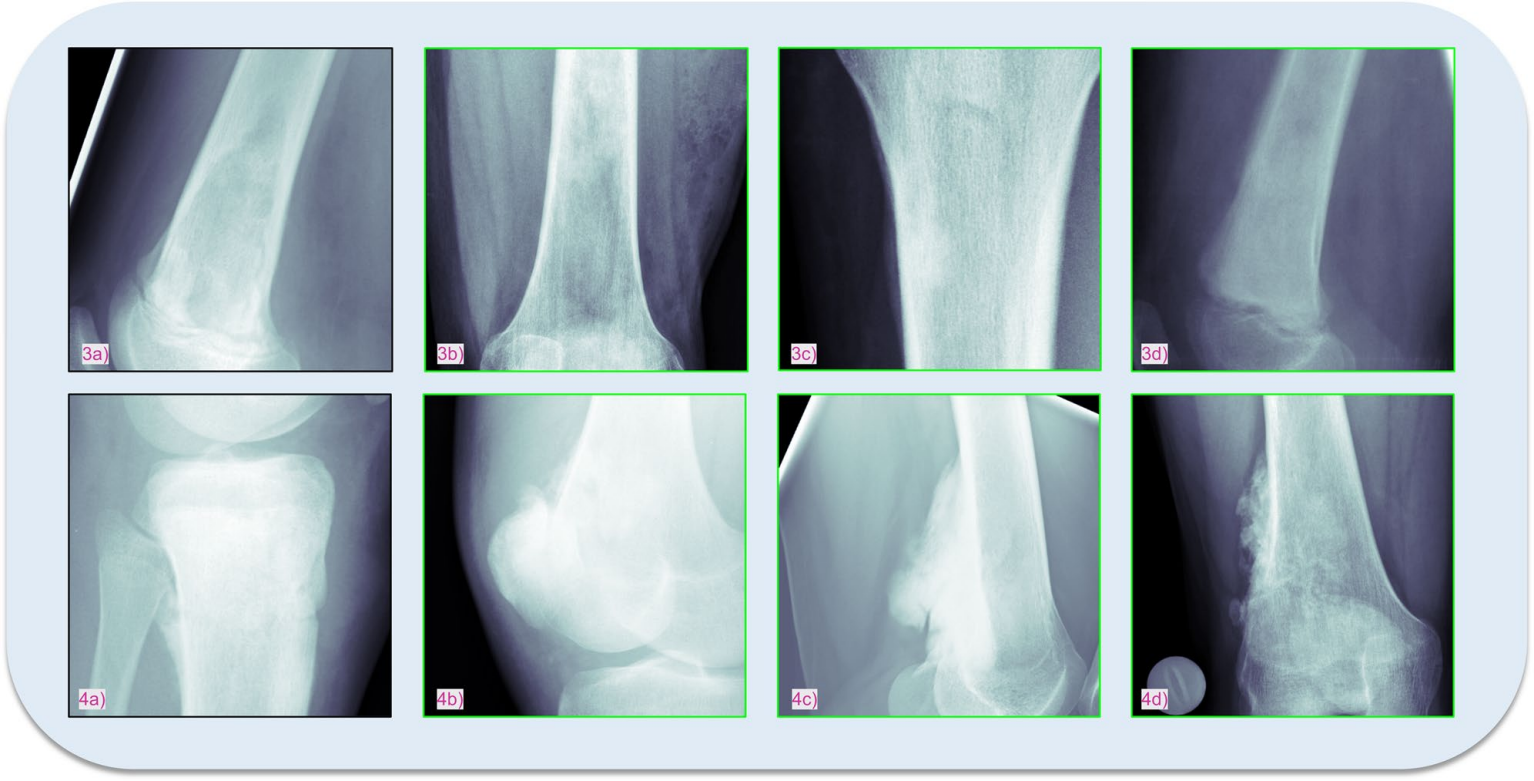
Examples of osteosarcoma X-rays illustrating the model’s effectiveness in clustering diverse manifestations of the same tumour entity. The target image is enclosed in a black frame, and correctly clustered images are indicated with a green frame

Initial Shapiro-Wilk tests were performed to assess the normality of the distribution of model performance metrics. The results suggested a normal distribution for most metrics, providing a basis for the use of parametric tests. Consequently, ANOVA was utilised to analyse the significance of differences in model performance, revealing significant disparities across the models (*p* < 0.0001 for all metrics, threshold at *p *= 0.05). Despite the limited sample sizes, ANOVA was considered appropriate due to the normality of the data and the robustness of this test under certain conditions. Following the ANOVA, Tukey’s HSD (Honestly Significant Difference) post hoc tests were conducted for pairwise model comparisons, which identified statistically significant differences, indicating that our approach significantly outperformed the baseline models (Table 3). Figures 5 and 6 show examples of correctly mapped osteochondromas and osteosarcomas from different patients and visually different appearances. The first images (1a, 2a, 3a, 4a - black) show the target images and the second to fourth images (1b-1d, 2b-2d, 3b-3d, 4b-4d - green) in each row show the correspondingly clustered images.

**Table 3:** Statistical significance of model performance metrics—ANOVA results demonstrate the overall significance of differences in accuracy, precision and recall among all tested models. Tukey’s HSD (Honestly Significant Difference) post hoc analysis further identifies the specific pairwise comparisons that are statistically significant. The *p* values indicate that the performance of ‘Our Approach’ is significantly different from both baseline models, and there is a significant difference in performance between the two baseline models

	**ANOVA**	**Tukey’s HSD post hoc test**
*p* values of test metrics		
Accuracy	< 0.0001	–
Precision	< 0.0001	–
Recall	< 0.0001	–
*p* values of model comparison		
Baseline 1: ResNet50 vs. Baseline 2: Transformer	–	0.0035
Our approach vs. Baseline 1: ResNet50	–	0.001
Our approach vs. Baseline 2: Transformer	–	0.001

## Discussion

The main result of this study was that we were able to develop an algorithm for real-time classification of ten preselected primary bone tumour entities that significantly outperformed a widely used [24] and a state-of-the-art model [25] and those shown in similar studies [17, 19] by circumventing the problem of confounding factors through clustering of the *k* most similar radiographs to a target image rather than classifying all different appearances and different anatomical structures of the same tumour pathology into one class. Further, identifying the most similar cases also allows large amounts of knowledge and experience lying dormant in clinical systems, such as previous diagnoses, treatments, etc., to be attributed to new and undiagnosed patients, potentially supporting an early and specific diagnosis. We hypothesise that the poor performance of the baseline models as well as the poor scores for recall across all approaches originate in overfitting due to limited available data and even more so because of significant class imbalances.

Similar studies were published [17, 19]. For example, von Schacky et al [17] presented a multitask DL model for simultaneous detection, segmentation and classification of bone lesions and compared the results with those of radiologists with different levels of experience. The general task of classifying bone lesions as well as the investigated entities are similar to our study. Their model achieved a classification accuracy of 43.2%, whereas a radiology resident achieved 44.1% and an MSK fellow radiologist 58.6% in classifying bone lesions by entity. While our metric scores are significantly higher, von Schacky et al had to cope with a lower ratio of samples per class. A major problem for the DL model probably was that bone tumour entities can occur in different anatomical regions and demonstrate different appearances. Therefore, a DL model has to classify the same pathology with different anatomical and visual features into the same class to predict correctly. As illustrated in Figs. 5 and 6, our model was able to bypass this issue by clustering only the *k* most similar cases and calculating the final prediction of the entity based on a majority vote. Their study underlines the complexity of precise identification of bone neoplasms for DL models as well as for human experts. Despite the widespread use of previous research projects analysing AI and humans in a direct comparison [16], the future use of AI to support instead of replacing medical experts is more likely. Another similar study was recently published by Kuanr et al [19]. The main concept behind their study was to identify similar COVID-19 patients based on comparably homogenous chest radiography by applying feature extraction accomplished by a DL model. The approach of comparing similar patients based on x-ray images is similar to that of our study. However, by implementing a majority vote on top of the clustered images for final metric calculation, we additionally demonstrated a classification for multiple entities and heterogenous pathologies. To the best of our knowledge, no study has yet shown a RS approach with majority vote to conclude in a classification of several bone tumour entities or link to previous sarcoma patient data.

The general approach of utilising retrospective datasets, training a DL model to extract meaningful image features and clustering similar cases based on imaging data with a nearest neighbour model is adaptable to other pathologies and scenarios as well. However, we hypothesise that the heterogeneity and multiple manifestations of bone tumours are one of the main reasons why we have achieved such a significant improvement with our algorithm compared to conventional classification approaches. It has been shown before that ensemble methods tend to give better results when the models and datasets have a large variety [29]. For tumour entities that occur more frequently in the same anatomic region, a classical approach would yield better results to begin with. Nevertheless, the concept of finding similar cases to compare with previous treatments of patients may be relevant to any other pathology.

The major limitation of this study is that we did not consider clinical data in the assessment of the tumour entity. Although plain radiographs are crucial for the initial screening for a possible bone tumour [5, 8, 30, 31], further classification requires the inclusion of clinical data (and possibly additional imaging) [9]. However, we hypothesise that some clinical information such as the patient's age, anatomical region, or tumour location is partially represented in the x-ray images and therefore indirectly integrated into our prediction model. Inclusion of clinical data and other bone tumour entities will be explored in future studies. Another limitation arises from the limited data set. While 1792 radiographs are a considerable number for the rare entities of MSK tumours, a mean of 179 samples per class is rather low in view of the heterogeneity of MSK lesions and additionally in the context of DL applications. Although approximately 10% of the data set consists of external radiographs from general practitioners, external radiologists, etc. uploaded to our clinical systems, another limitation is that the model needs to be tested on external data to further assess generalisability [32] before suitability for clinical use can be evaluated [33]. Although we managed to circumvent problems with confounding factors, the fact that most of the data were collected in a single centre could still affect the robustness of the model: different image characteristics associated with different radiographic devices or different patient characteristics could cause this.

In conclusion, we have demonstrated a way to deal with limited data and complex classification problems, providing a real-time feedback for bone tumour assessment. The proposed framework can link undiagnosed patients with previous experience and knowledge lying dormant in our clinical systems. Additionally, we have used AI methodology to leverage previously collected knowledge based on previous patient journeys, allowing us to draw on human experts to potentially assist general practitioners and young physicians in difficult situations and enable early and specific diagnosis.

## Supplementary Information

Below is the link to the electronic supplementary material.

Supplementary file1 (PDF 307 KB)

## Abbreviations

AI: Artificial intelligence
DL: Deep learning
LSH: Locality-sensitive hashing
RS: Recommender system

## Declarations

### Guarantor

The scientific guarantor of this publication is Daniel Rueckert.

### Statistics and biometry

One of the authors has significant statistical expertise.

No complex statistical methods were necessary for this paper.

### Study subjects or cohorts overlap

No study subjects or cohort overlap has been reported.

### Methodology

• retrospective

• diagnostic or prognostic study

• performed at one institution
