# Supplementary material for: Recommender-based bone tumour classification with radiographs—a link to the past
Source: Eur Radiol. 2024 Mar 15;34(10):6629–38. doi: 10.1007/s00330-024-10672-0 (PMC11399296; doi:10.1007/s00330-024-10672-0)

Table 1: Shapiro-Wilk normality test results for model performance metrics - This table summarizes the results of the Shapiro-Wilk normality tests conducted on the model performance metrics accuracy, precision, and recall for each model (ResNet50, Transformer, and Our Approach). The table presents both the Shapiro-Wilk statistic and the corresponding p-values, providing insights into the distribution characteristics of the data for each metric across the models.

**Metric**	**Shapiro-Wilk Statistic**	**P-Value**
ResNet50 Accuracy	0.93	0.60
Transformer Accuracy	0.98	0.91
Our Approach Accuracy	1.00	0.94
ResNet50 Precision	0.89	0.35
Transformer Precision	0.87	0.25
Our Approach Precision	0.89	0.35
ResNet50 Recall	0.80	0.09
Transformer Recall	0.93	0.60
Our Approach Recall	0.76	**0.03**

Supplementary Figure 1: Display of included pathologies: 1a) aneurysmal bone cyst (ABC), 1b) chondroblastoma, 1c) chondrosarcoma, 1d) enchondroma, 1e) Ewing sarcoma, 1f) fibrous dysplasia, 1g) giant cell tumour, 1h) non-ossifying fibroma (NOF), 1i) osteochondroma, 1j) osteosarcoma.

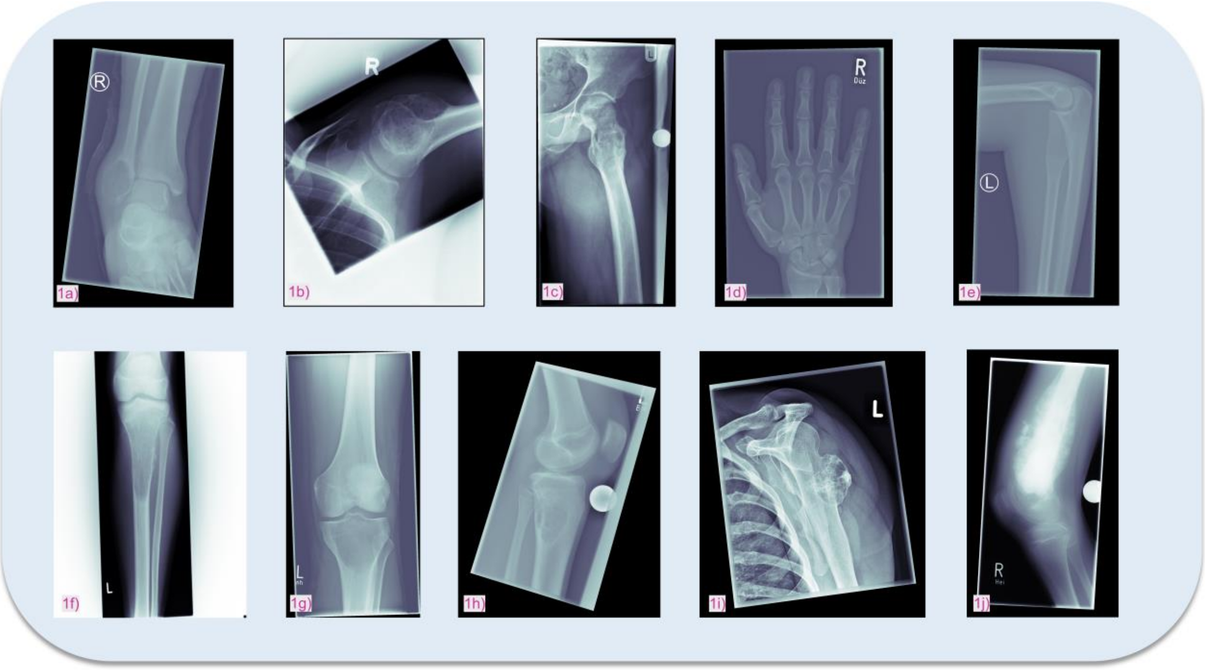

Supplementary Figure 2: Display of Enchondroma X-rays, showcasing the model's clustering accuracy for varied appearances of this tumour entity. The target image is outlined in black. Correctly matched images are indicated with a green frame, and an incorrectly clustered image (Chondrosarcoma, 3b) is highlighted with a red frame.

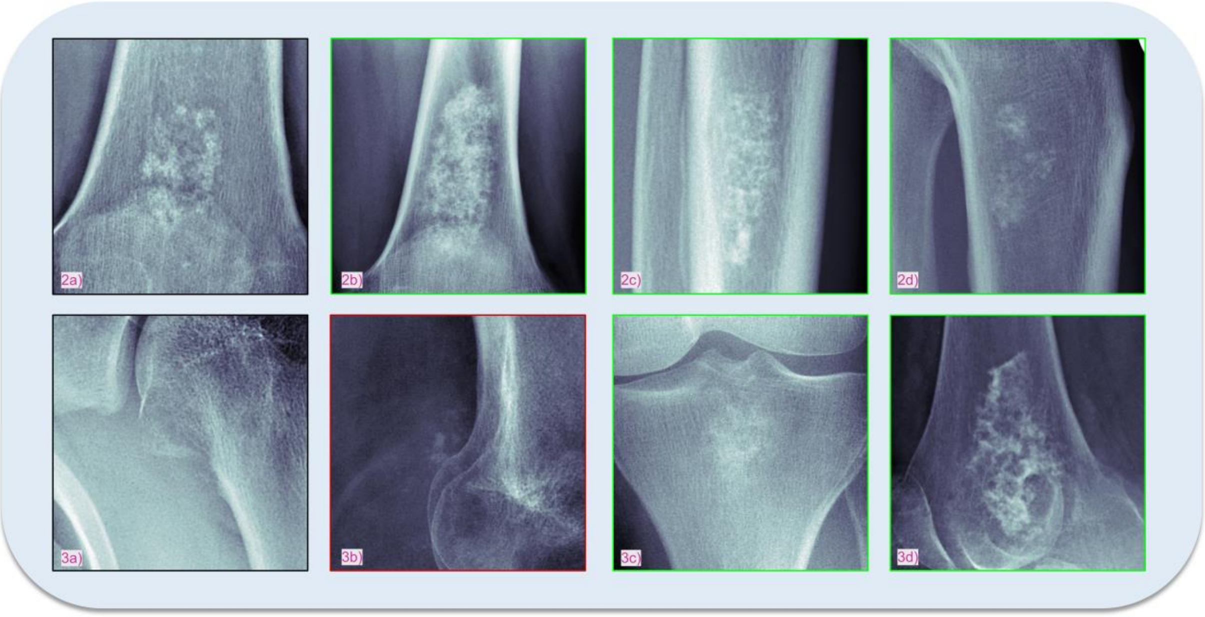

Supplementary Figure 3: Presentation of Chondrosarcoma X-rays, illustrating the model's effectiveness in accurately clustering different manifestations of this tumour entity. The target image is framed in black, correctly clustered images in green, and an incorrectly matched image (Osteochondroma, 5c) in red.

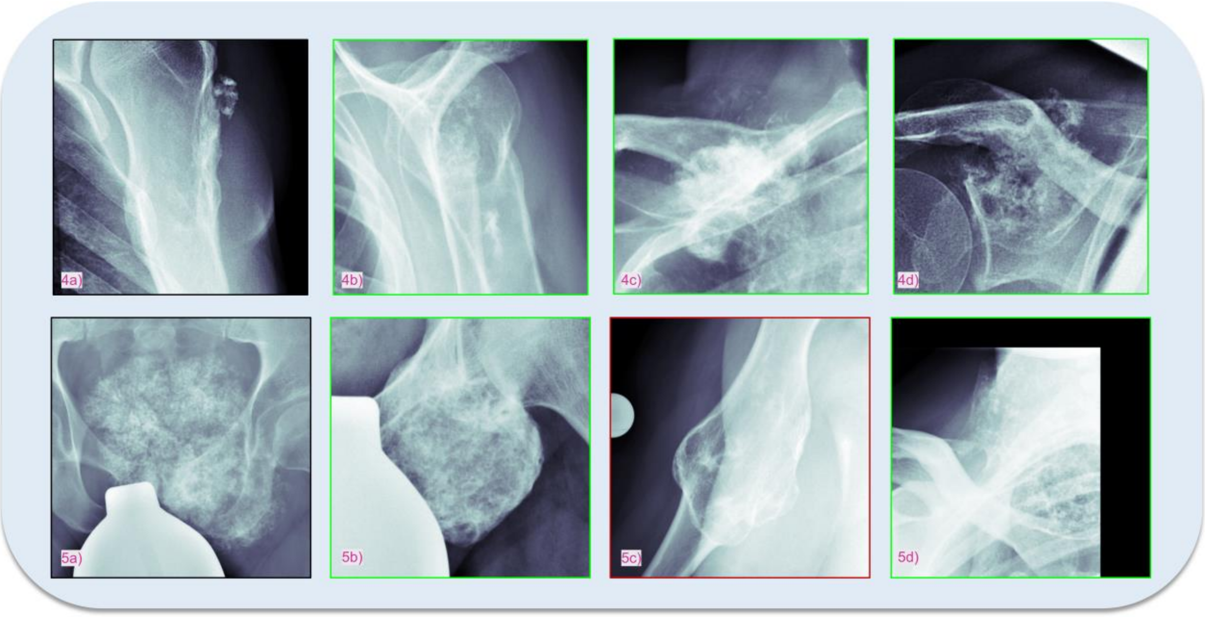

Supplement: Supplementary file 1 — Supplementary file1 (PDF 307 KB) [file 330_2024_10672_MOESM1_ESM.pdf]
